# Antioxidant Effects of Brown Algae Sargassum on Sperm Parameters: CONSORT-Compliant Article

**DOI:** 10.1097/MD.0000000000001938

**Published:** 2015-12-31

**Authors:** Alireza Sobhani, Tasnim Eghbal Eftekhaari, Mohammad Esmaeil Shahrzad, Mohammad Natami, Soghra Fallahi

**Affiliations:** From the Pathology Department, Shariati Hospital (AS); Molecular Medicine Research Center (TEE); Student Research Committee (MES); Department of Urology (MN); and Hormozgan Fertility & Infertility Research Center (SF), Hormozgan University of Medical Sciences, Bandar Abbas, Iran.

## Abstract

The occurrence of oxidative stress during the sperm freeze-thaw cycles affects the sperm parameters and eventually leads to a decrease in its reproductive potential. Sperm protection against oxidative reactions during freezing is done by antioxidants. Since the selection of a suitable sperm cryopreservation bank is effective in maintaining acceptable reproductive potential and motility of sperm during cryopreservation.

This study aimed to evaluate the antioxidant effects of different doses of the extract of brown algae Sargassum on oxidative stress and frozen human sperm parameters.

We conducted a randomized controlled trial on the semen samples from 11 healthy men in the age group of 25 to 36 years. The samples were collected by masturbation after 3 to 5 days of abstinence from ejaculation. The specimens were divided into 3 equal parts, including 1 control group and 2 experimental groups.

The 2 experimental groups were frozen using the rapid solidification technique with Sargassum extract at doses of 250 and 500 μg/mL.

Motility and morphology of sperms were measured using a computer system and CASA software and the amount of reactive oxygen species was determined using Oxisperm kit.

Sargassum extract significantly decreased the amount of reactive oxygen species (*P < *0.005) and at doses of 250 and 500 μg/mL, significantly increased the overall motility (*P < *0.006) and progressive motility (*P < *0.007) after solidification, but did not affect the normal morphology of sperms.

The addition of ethanol extract of Sargassum prevents reactive oxygen species production during the solidification process and improves sperm motility at doses of 250 and 500 μg/mL.

## INTRODUCTION

It is well-known that Marine algae, such as Brown algae Sargassum, are rich sources of antioxidant compounds.^1^ The antioxidant properties of this species can be used to neutralize the effects of oxidative reactions in several processes such as cryopreservation, which is a branch of cryobiology that deals with the long-term preservation of cells at very low temperatures.^2,3^ Cryopreservation is usually used to increase the success of assisted reproductive techniques by preparation of sperm bank for men undergoing chemotherapy, radiotherapy, and testicular surgery or with ejaculatory failure.^4,5^ However, semen is exposed to cold shock and osmotic pressure during cryopreservation resulting in an increase in the oxidation rate of the membrane due to the greater percentage of oxidative reactions and excessive production of reactive oxygen species (ROS), which ultimately reduces sperm motility, life, and reproductive capacity ^6,7^ as a consequence of damage to the cell membrane and the morphological abnormalities such as twisted tails, damage to the acrosome, DNA fragmentation, and decreased sperm functions.^8^ Therefore, a suitable cryopreservation bank should be selected to maintain the acceptable reproductive potential and motility of sperms during the cryopreservation.^9^ Antioxidants have reverse functions to reduce the effects of ROS, including reducing the oxygen concentration, eliminating the catalytic metal ions, and harvesting a variety of free radicals such as superoxide (O_2_^−^), hydroxyl (OH), and hydrogen peroxide (H_2_O_2_).^10^ In this regard, recent studies showed that during the cryopreservation, the sperm plasma membrane can be protected by administration of antioxidants against oxidative reactions,^11^ as the cytoplasm of sperm has a low content of enzymatic antioxidants and it is dependent on the antioxidant defense of semen,^12^ which is removed during the processing of semen and increase the sensitivity of sperm against oxidative stress.^13^ Therefore, the addition of external antioxidants to sperm can be used to compensate the lack of defensive shield.^14^ As mentioned above, Brown algae Sargassum was shown a high antioxidant power in vitro due to having phenolic compounds.^2^ In fact, phlorotannins are the major phenolic compounds of brown algae with an antioxidant role.^15^ It was reported that the aqueous extract of Sargassum significantly inhibits lipid peroxidation at a concentration of 800 μg/mL. The extract also has a high capacity for the removal of OH and 1,1-diphenyl-2-picrylhydrazyl (DPPH) free radicals and can inhibit glutathione *S*-transferases (GST). These effects increase with the elevation in concentration of the extract.^16^ Considering the possible antioxidant effects of the extract of Sargassum and the fact that no studies were conducted on the effects of this extract on human semen parameters, this study investigated the effects of concentrations of 250 and 500 μg/mL of Sargassum extract on the amount of reactive oxygen species (ROS) and human sperm parameters after cryopreservation.

## MATERIALS AND METHODS

### Sargassum Extract Preparation

Brown algae *Sargassum angustifolium* were collected from the shores of Qeshm Island in the Persian Gulf. They were washed in seawater to remove epiphytes and transported to the laboratory as soon as possible. In the laboratory, the algae were dried under shade after being washed with distilled water. Thereafter, the algae were powdered and soaked in methanol for 24 h and then, the extract was separated from algae powder using filter paper and algae powder was soaked in methanol for 24 h again. The resulting extract was dried by the rotary evaporator and again dissolved in methanol at a concentration of 0.5 mg/mL.

### Semen Sample Collection

The required semen samples were collected from healthy individuals in the age group of 25 to 36 years without varicocele, renal disease, hepatic disease, hematological disease, hormonal disorders, genetic disorders, erectile dysfunction, infection, testicular trauma, and so on. It was conducted by masturbation after 3 to 5 days of abstinence from ejaculation. Ethical approval was not required as all the procedures were in vitro and no intervention was conducted on humans. Written informed consent was collected from all subjects before beginning of the study and those not consenting to participate in the study were ruled out. Immediately after receiving the samples, initial semen analysis was performed on a part of it and samples that were normal in terms of World Health Organization (WHO) 2010 criteria (sperm count ≥ 15 million/mL, total motility ≥ 40%, progressive forward motility ≥ 32%, normal morphology ≥ 40%, seminal volume 1.5 mL, pH ≥ 7.2, normal appearance and viscosity, and maximum liquefaction time of 1 h at room temperature) were selected which were finally 11 samples. The remaining of the semen samples from selected individuals was used for washing, cryopreservation, and determination of the level of oxidative stress.

### Sperm Preparation (Washing and Freezing)

In order to prepare the sperms for cryopreservation, the remaining of the semen samples were divided into 3 groups: (1) cryopreservation control group, (2) cryopreservation group with a dose of 250 μg/mL of Sargassum extract, and (3) cryopreservation group with a dose of 500 μg/mL of Sargassum extract. The sperm washing was performed using the swim-up method and applied 3 times on each group. The equal volume of semen sample and human tubal fluid (HTF containing 5% albumin) was centrifuged at 2000 rpm for 10 min. Then, the supernatant plasma was discarded and the same volume of HTF as the remaining sediment was added again and centrifuged. The third time sediment was again mixed with HTF and then, the commercial sperm freezing medium (HEPES containing 10% albumin) was added slowly and for the second and third groups, Sargassum extract was added at specified concentrations. Some of the mixture of each group was frozen in straws and the 2 sides of the straws were sealed with flame. The straws containing semen and freezing medium were stored in nitrogen vapor-phase for ∼10 min before being transferred to liquid nitrogen. They were transferred to the nitrogen tank to complete the freezing process after formation of frost around the straws. After a week, the straws were stored at room temperature for 10 to 30 min for the frosts to melt and then incubated for complete liquefaction. Then, 1 side of the straw was opened and the straw contents were discharged. The microscopic parameters of semen were examined using Computer Assisted Sperm Analysis (CASA) system and the oxidative stress levels through Oxisperm kit in all the 3 cryopreservation groups.

### Evaluation of Oxidative Stress Levels

To determine the level of oxidative stress occurred in semen, commercial Oxisperm kit was used. The test was performed according to the kit instructions. First, the tube containing the reaction gel was placed in a 900 watts microwave for 1 min for liquefaction. After the gel was cooled down to 37°C, it was mixed with semen sample and incubated at 37°C for 45 min. After the required time, the color of sediment was compared with the colors noted in the kit manual, which varies based on superoxide anion concentration from pale pink to dark purple at 4 levels of N1, N2, N3, and N4.

### Sperm Motility Determination Using Computer-Assisted Sperm Analysis (CASA) System

Twenty micro liters of the homogenized semen was placed on a clean slide by sampler and cover slip was put on it and observed under a microscope. Then, waited ∼60 s for the sperms to lose their turbulence and stay steady. CASA system is composed of a microscope equipped with a camera and connected to a computer which the V.T sperm (2.1) software is already installed on it. The software first detects the sperms based on their size and elliptical or circular shape and then records their movement for 1 s and calculates the motility parameters, including grade A: percentage of sperms with fast forward motility, grade B: percentage of sperms with slow forward motility, grade C: percentage of sperms with nonprogressive motility, grade D: percentage of sperms with no motility, progressive motility (A + B) and general motility (A + B + C).

### Determination of Sperm Morphology Using Diff-Quick Staining Technique and CASA System

For this purpose, 200 λ of semen sample was mixed with normal saline solution in a tube and centrifuged for 5 min at 2000 rpm. A slide was prepared from the homogenized sediment and after drying it was placed in solution number 1 of the coloring kit (fixation) for 15 to 20 s and then in color solution number 2 for 15 to 20 s and again 15 to 20 s in solution number 3 of the kit. The slide was washed with distilled water and dried. Normal or abnormal sperm morphology was evaluated in the prepared slides using the 100× lens and immersion oil by the CASA system.

### Statistical Analysis

Statistical analysis was performed using SPSS 19 and descriptive and inferential statistics methods. The results were presented as mean ± standard deviation. Repeated measures were used for analysis of the quantitative data. Kendall's correlation coefficient was used for analysis of the qualitative data. Furthermore, the Bonferroni post hoc test was used for pairwise comparison of groups in the case of presence of significant difference. Significance level was considered *P* < 0.05.

## RESULTS

### Comparison of the Sperm's Mean Motility

**FIGURE 1:**
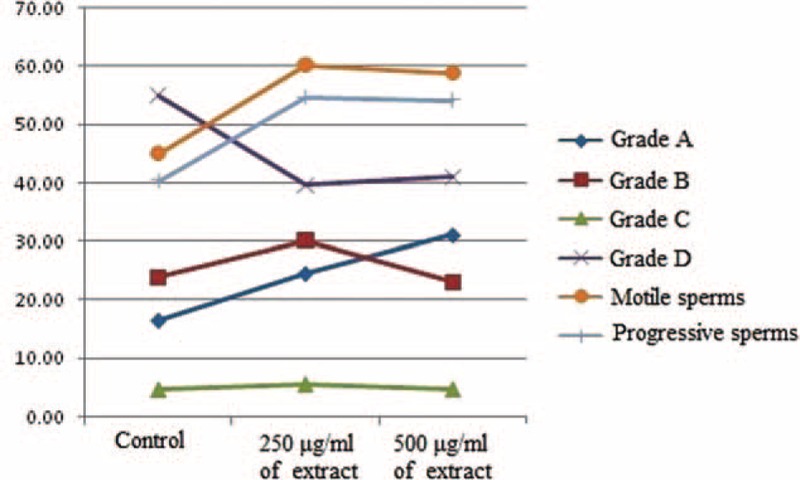
Comparison of sperm motility between the groups treated with different concentrations of extract and the cryopreservation control group.

As shown in Table 1, concentrations of 250 μg/mL and 500 μg/mL of the Sargassum extract significantly improved the general motility and progressive motility of sperms in freezing environments in comparison with the cryopreservation control group (*P* < 0.05). However, in Grade A motility (fast forward motility), significant increase was only observed in concentration of 500 μg/mL of Sargassum extract compared with the control group (*P* < 0.05). Moreover, a significant decrease occurred in the percentage of sperms with no motility in both 250 μg/mL and 500 μg/mL concentrations of the extract (*P* < 0.05). No significant differences were observed in grades B and C between the extract containing and control cryopreservation groups (*P* > 0.05). Figure 1 also summarized changes in the average motility of all the 6 mentioned variables.

**TABLE 1:**
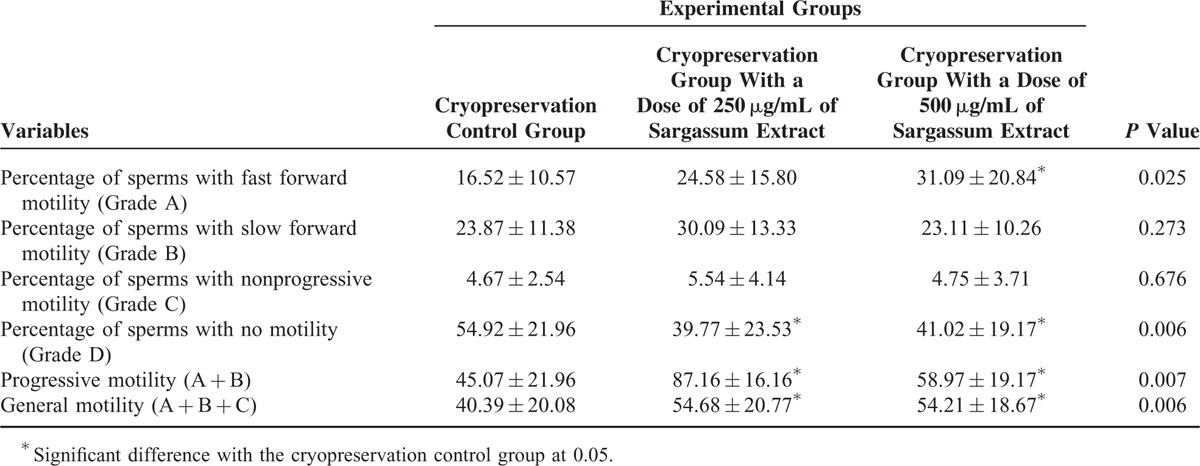
The Comparison of Average Motility in Different Grade Sperms Between the Experimental Groups

### Mean Comparison of the Normal Sperm Morphology

**FIGURE 2:**
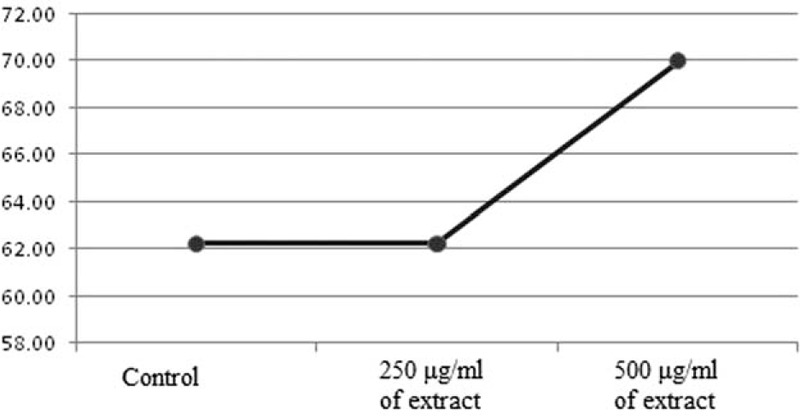
Comparison of the average percentage of normal sperm morphology between the groups treated with different concentrations of extract and the cryopreservation control group.

According to Table 2, the mean comparison of normal sperm morphology determines that there is no significant difference between the extract containing and control cryopreservation groups (*P* > 0.05). Although there is an increase in the percentage of normal morphology at the dose of 500 of the extract, it is not statistically significant. Figure 2 indicates the average percentage of normal sperm morphology in all 3 experimental groups.

**TABLE 2:**
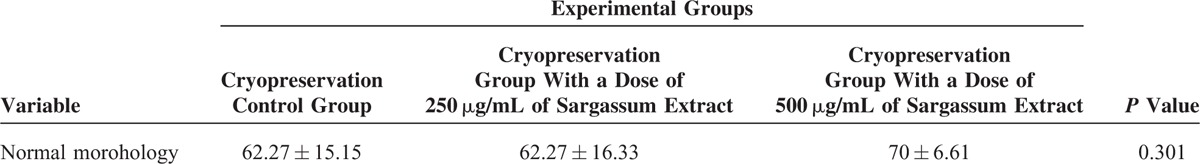
The Comparison of Average Percentage of Sperms With Normal Morphology Between the Experimental Groups

### ROS Concentrations and Oxidative Stress Rate

Table 3 shows the occurrence rate of oxidative stress in terms of superoxide anion concentration (ROS) in the experimental groups. Investigating the data shows that in the presence of Sargassum extract the frequency of N4, which represents the highest degree of oxidative stress, is zero and the frequency of N3 is also reduced compared with the cryopreservation control group. According to Kendall coefficient 0.408 calculated from the table, *P* value was evaluated 0.005; therefore, it can be concluded that there is a significant relationship between the intensity of oxidative stress and the experimental groups. Thus, by increasing the dose of extract, the intensity of oxidative stress is reduced due to neutralization effect of Sargassum extract.

**TABLE 3:**
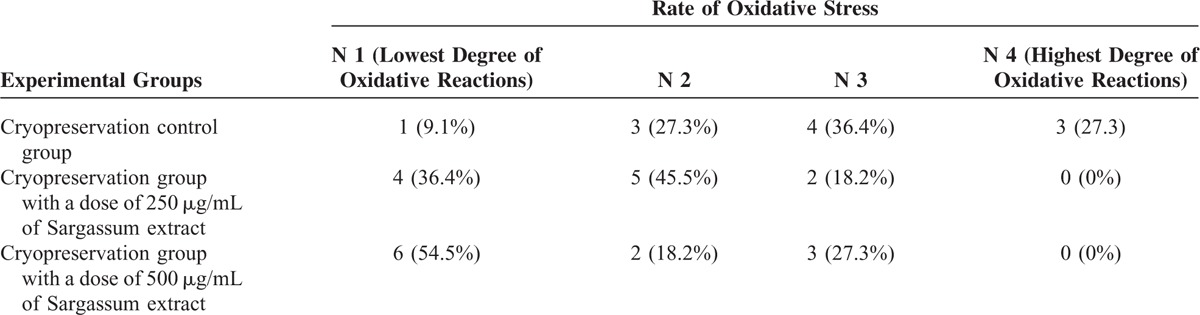
The Comparison of the Occurrence Rate of Oxidative Stress in Different Cryopreservation Groups

## DISCUSSION

Cryopreservation leads to excessive production of reactive oxygen species (ROS) that can reduce the sperm reproductive potential by affecting determinants.^7,17,18^ As mentioned, the sperm is dependent on the extracellular antioxidant systems, especially semen plasma, to overcome oxidative reactions.^19^ However, the semen plasma is separated and discarded during the sperm processing which increase the sensitivity of sperms against oxidants.^20^ Therefore, many researchers were attracted to provide a source of antioxidants to protects sperms during processing.^21^ In recent years, they revealed the role of plants with antioxidant properties in improving the quality of sperm parameters.^22^ However, to the best of our knowledge no studies have reported the effects of Sargassum antioxidants on sperms parameters, so far. Interestingly, findings of the present experimental study illustrated the positive effects of alcohol extract of Sargassum on the reduction of oxygen-free radicals and the intensity of oxidative stress and also the elevation in percentage of human sperms motility. Therefore, our report is consistent with the results of other researchers including Patra et al,^16^ Ye et al,^23^ and Lim et al^24^ who stated that the Sargassum extract inhibits the free radicals. In the present study, the effect of Sargassum extract on semen parameters revealed significant differences in fast forward motility, total motility, and progressive motility at doses of 250 and 500 (μg/mL) of the extract compared with the cryopreservation control group. Related to this study, the use of antioxidants in pomegranate juice resulted in an improvement in sperm motility in the test group compared to the control group.^25^ Similar findings were also reported after freezing with melatonin ^26^ as well as in the study by Bansal with a dose of 5 mM of glutathione, which are consistent with the results of the present study.^27^ To justify the findings of present survey, we should refer to the studies in which they detected antioxidants in the brown algae Sargassum including the phenolic compounds, flavonols or flavonol glycerol in this species of algae.^2,15,28,29^ Indeed, the phenol rings of polyphenols found in Sargassum are strong chelators of heavy metals that can lead to elimination of free radicals by pulling electrons.^30^ On the other hand, substances such as flavonoids were reported to perform their antioxidant activity by increasing the levels of glutathione peroxidase which is the enzymatic antioxidant present in the sperm.^31^ Therefore, the final result of both mechanisms is the reduction in the amount of oxygen-free radicals, which is responsible for the increase in the sperm motility.^32^

In the present study, none of the doses of the Sargassum extract could increase the percentage of normal sperm morphology. Similar results were obtained by Jang et al in 2006 which indicated that the addition of antioxidant taurine failed to improve the normal sperm morphology.^33^ Another study in 2013 regarding the antioxidant effect of curcumin on rat sperm morphology, also revealed no significant correlation.^34^ In contrast to these findings, Bucak et al reported the significant effects of inositol (as an antioxidant substance) on the structural integrity of acrosome and sperm morphology.^35^ These differences may be due to the conditions of experiment. Indeed, antioxidants in vitro cannot cause changes in the sperm morphology. To confirm this hypothesis, we can refer to the study in which pomegranate juice oral administration (as an antioxidants) by rats could significantly improve sperm morphology.^36^ Similarly, administration of the onion extract were observed to be effective on the number of Sertoli cells, the percentage of sperms with normal morphology and epididymal weight.^37^ Finally, Heidary et al in 2008 studied the effect of consumption of saffron (*Crocus sativus*) on the sperm parameters in infertile men and indicated that taking this herb for 3 months is effective in improving the sperm morphology.^38^

## CONCLUSION

In conclusion, we can say that maintaining the sperm parameters after cryopreservation stages is definitely effective on the fertilization success rate. On the other hand, the survival of sperm and its parameters after freezing depends on several factors such as the culture medium elements. In this regard, standardization of laboratory culture media and creating optimum conditions to maintain the sperm parameters are the main issues. Therefore, it seems necessary to find supplement substances in order to create favorable conditions for the preservation of these parameters. In the present study, regarding the positive effect of Sargassum extract on the elimination of free radicals and the significant increase in the percentage of sperm motility and the significance of these parameters in fertility, the negative effects of freezing on sperm parameters can be reduced by adding the extract of Sargassum to the culture medium.
