# Antioxidant Effects of Brown Algae Sargassumon Sperm Parameters: CONSORT-Compliant Article: Erratum

**DOI:** 10.1097/01.md.0000481323.52215.34

**Published:** 2016-03-03

In the article “Antioxidant Effects of Brown Algae Sargassumon Sperm Parameters: CONSORT-Compliant Article”,^1^ which appeared in Volume 94, Issue 52 of *Medicine*, Dr. Eftekhaari's degree and affiliation were originally presented incorrectly. The article has since been corrected online.
